# Massive Point Cloud Processing for Efficient Construction Quality Inspection and Control

**DOI:** 10.3390/s24216806

**Published:** 2024-10-23

**Authors:** Zhansheng Liu, Zehong Liu, Zhe Sun

**Affiliations:** 1 College of Architecture, Civil and Transportation Engineering, Beijing University of Technology, Beijing 100124, China; liuzhansheng@bjut.edu.cn (Z.L.); s202264496@emails.bjut.edu.cn (Z.L.); 2 The Key Laboratory of Urban Security and Disaster Engineering of the Ministry of Education, Beijing University of Technology, Beijing 100124, China; 3 Chongqing Research Institute of Beijing University of Technology, Chongqing 401121, China

**Keywords:** spatiotemporal data, point cloud data, data processing policy, construction quality control

## Abstract

The construction of large-scale civil infrastructures requires massive spatiotemporal data to support the management and control of scheduling, quality control, and safety monitoring. Existing artificial-intelligence-based data processing algorithms rely heavily on experienced engineers to adjust the parameters of data processing, which is inefficient and time-consuming when dealing with huge datasets. Limited studies have compared the performance of different algorithms on a unified dataset. This study proposes a framework and evaluation system for comparing different data processing policies for processing huge spatiotemporal data in construction quality control. The proposed method compares the combination of multiple types of algorithms involved in the processing of massive point cloud data. The performance of data processing strategies is evaluated through this framework, and the optimal point cloud processing strategies are explored based on registration accuracy and data fidelity. Results show that a reasonable choice of combinations of point cloud sampling, filtering, and registration algorithms can significantly improve the efficiency of point cloud data processing and satisfy engineering demands for data accuracy and completeness. The proposed method can be applied to the civil engineering problem of processing a large amount of point cloud data and selecting the optimal processing method.

## 1. Introduction

In this era of rapid technological advancement, three-dimensional spatial data have become an indispensable resource in multiple fields, including urban planning, environmental monitoring, and civil construction. As the core content of 3D spatial data, the accuracy of spatiotemporal data processing and analysis directly affects the effect and quality of the application. With advancements in data acquisition technologies, especially the widespread use of laser scanning and photogrammetry, the volume of spatiotemporal data has increased dramatically. A pressing issue now arises: how to maintain processing efficiency without sacrificing the precision of the data. Among the various point cloud processing methods, point cloud sampling, filtering, and registration algorithms are three fundamental and critical steps. Point cloud sampling, as a means to reduce data volume, can significantly enhance the efficiency of subsequent processing steps; point cloud filtering is crucial for data quality, directly affecting the accuracy of subsequent applications; and point cloud registration is the cornerstone of building complete three-dimensional models. Although numerous studies have been conducted to explore the application of various algorithms in a single scene, few studies have systematically compared the performance of different algorithms on the same dataset, and there is even a gap in the research on large-scale point cloud data processing.

This study focuses on original point cloud data from two stations employing three types of subsampling algorithms (Octree, Space, Random) and three filtering algorithms (Gauss, Radius, Statistical), combined with three registration algorithms (ICP, PFH, FPFH) to construct a comparative framework comprising 81 different combinations. Through this framework, we can not only evaluate the performance of each algorithm combination but also explore the optimal point cloud processing flow based on registration accuracy and data fidelity. This systematic comparison is of great significance for understanding different algorithms’ mechanisms and application scenarios in point cloud processing.

The remainder of this study is structured as follows. Section 2 provides a comprehensive review and analysis of point cloud data processing in civil engineering. Section 3 establishes the comparative framework for point cloud processing algorithms and introduces the different algorithms. Section 4 details the parameter selection for various point cloud processing procedures. Section 5 analyzes and compares the results of the point cloud data processing algorithm combinations. Finally, the main points of this study are summarized.

## 2. Research on Point Cloud Data in Construction Quality Inspection

In the fields of architecture and civil engineering, the application of spatiotemporal data is becoming increasingly important. By scanning buildings, terrain, and other structures in 3D, it is possible to create highly accurate digital twin models, which can be useful in several phases such as design, construction, supervision, and maintenance.

In the construction industry, spatiotemporal data are generated using a variety of sensors and technologies for architectural and infrastructure-related applications, including 3D laser scanning [1], photogrammetry [2], video measurement [3], drones [4], and stereoscopic cameras [5]. Three-dimensional scanning is used to acquire and store captured point cloud data, where data points are defined by X, Y, and Z coordinates and typically contain color, classification values, intensity values, time, and more. Point cloud data need to be processed to realize the theoretical benefits of the point cloud in terms of productivity, quality, and safety enhancement in construction and infrastructure [6,7]. Aijazi et al. [8] proposed the feasibility of robust 3D point cloud registration in positioning and mapping applications using a Spirograph-type non-repetitive scanning mode lidar sensor. Denayer et al. [9] provided an in-depth comparison of six registration methods for 3D scanning, focusing on classical techniques and deep-learning-based solutions.

In the field of spatiotemporal data applications, many scholars have attempted to combine spatiotemporal data with various technologies for tasks such as building reconstruction and construction quality control. Son et al. [10] reviewed the use of point cloud data in two specific applications: production monitoring and automated layout of civil infrastructure. Liu J et al. [11] summarized the methods of combining 3D laser scanning with BIM and provided a comprehensive review of its applications throughout the building lifecycle. Tang et al. [12] reviewed the technologies related to the automatic reconstruction of completed models from scanned point cloud data. Wang Q et al. [13] reviewed the applications of 3D point cloud data in the construction industry from 2004 to 2018, focusing mainly on the areas of 3D model reconstruction and geometric quality inspection, but also including construction progress tracking, building performance analysis, construction safety management, and construction automation.

Spatiotemporal data acquisition techniques are becoming more mature, and the demand for 3D point cloud data acquisition of existing buildings and infrastructures during the construction phase is increasing. Kim P et al. [14] proposed a new method for automatic three-dimensional point cloud registration in construction sites without the use of any manual targets. Point cloud data can be captured by scanning the existing conditions of building facilities to establish corresponding point cloud models. Bosché F [15] introduced a method for automatically tracking the completion status of construction sites using lasers and 3D CAD modeling, improving the Iterative Closest Point (ICP) algorithm to enhance registration quality.

In the area of spatiotemporal data processing and the combined application of algorithms, Hazer A. et al. [16] analyzed the use of deep learning techniques and algorithms in point cloud processing, including 3D object classification and segmentation as well as 3D object detection. Sun Z et al. [17] manually compared changes in point cloud data detected by two sets of 3D scans and inspection reports, identifying actual deformations in bridges, and proposed that automated integration analysis of image data and bridge inspection reports is technically feasible. Addressing the issue of massive spatiotemporal data from large-scale building scans being unsuitable for subsequent processing, Chen J et al. [18] proposed a point cloud compression method based on geometric complexity, retaining more geometric information while meeting compression ratio requirements. This method achieved a 30% compression ratio while ensuring minimal loss of geometric information. Shirowzhan et al. [19] conducted a comprehensive evaluation of building change detection algorithms, including pixel-level and point-cloud-level algorithms, and proposed two assessment criteria; this study has been significant in guiding building change monitoring. Although spatiotemporal data registration technology is relatively mature, an objective evaluation system is still needed to provide a quantitative analysis of different methods and promote high-quality registration techniques [20]. On this basis, Gu X et al. [21] reviewed point cloud registration algorithms, providing preliminary references for selecting appropriate algorithms in different scenarios. Table 1 displays the research of some scholars in the field of construction on spatiotemporal data.

Previous studies have proposed semi-automatic [22] or automatic methods [23] to apply scanning data to specific engineering fields and scenarios [24]. Although 3D laser scanning technology has significant advantages in related areas, the need for manual processing and training using large volumes of data after scanning consumes considerable time, which severely hampers its widespread application in civil engineering. There is a great need for a method that can reduce the amount of point cloud data and increase the efficiency and accuracy of point cloud data processing. Therefore, this study constructs a comparative framework for data processing algorithms based on traditional point cloud data processing methods. Through this framework, not only can the performance of each algorithm combination be evaluated, but the optimal point cloud data processing workflow can also be explored based on registration accuracy and data fidelity.

**Table 1:** Point cloud data-related studies.

Authors	Topic
Lu Q et al. [25]	Focused on automatic methods for generating point cloud processing for Building Information Modeling (BIM).
Truong-Hong et al. [26]	A method for automatically processing point cloud data and exporting CAD models suitable for subsequent structural calculations was proposed.
Kim M, Lee D. [27]	A framework for reconstructing digital models of buildings into 2D geometries using 3D point cloud data was proposed. The framework aims to generate 2D digital models quickly and efficiently by first discretizing the registered 3D point cloud into voxels and effectively removing outliers.
Liu Z et al. [28]	Researchers established a point cloud model by 3D scanning and proposed an intelligent construction method based on digital twins.
Kavaliauskas et al. [29]	Proposed a construction progress monitoring method based on the alignment of point cloud data with IFC and automatic object detection; performed automatic object detection by calculating the relationship between points and objects in the aligned data.
Zhang H et al. [30]	A three-step computational framework was proposed for semi-automatic detection of spalling and quantification of critical properties of reinforced concrete columns in PCD.
Fontana S et al. [31]	A new benchmark was proposed for point cloud registration algorithms, whose main goal is to allow rigorous comparisons between different methods.
Wang Q et al. [32]	Different 3D point cloud data acquisition methods were compared, and different processing methods and algorithms for data cleaning, data alignment, data segmentation, and object recognition were compared in detail.

## 3. A Framework for Comparing Point Cloud Data Processing Policies in Construction Quality Control

This study presents a framework for combining point cloud data processing algorithms and describes the algorithms used. Two sets of point cloud data were selected for processing. The combination of point cloud data processing algorithms consisted of three main stages: point cloud sampling, filtering, and registration. For each stage, three different algorithms were selected. Since each stage allows for 3 algorithm choices, the total number of possible combinations was calculated as 3 (sampling) × 3 (filtering) × 3 (registration) = 27 combinations per dataset. In the filtering stage, the two-site cloud data could be randomly combined between the three algorithms. Therefore, processing two sets of point cloud data resulted in a total of 27 × 3 = 81 possible algorithm combinations.

### 3.1. Point Cloud Data Processing Framework

**Figure 1:**
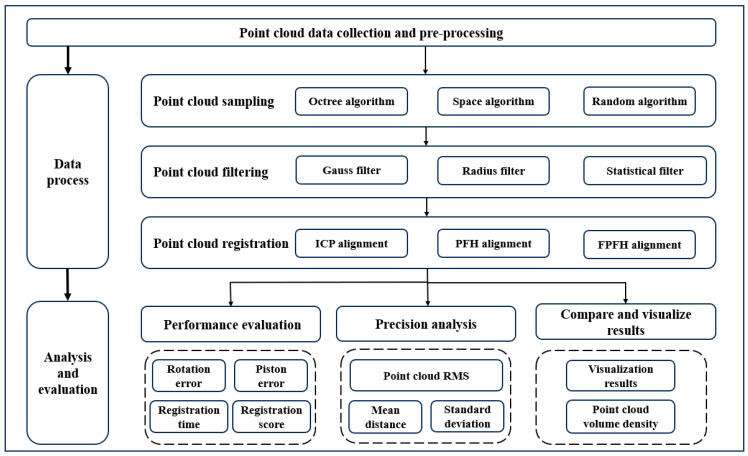
Point cloud data processing framework.

Three-dimensional laser scanning equipment was used to acquire point cloud data for preliminary preprocessing, dividing the point cloud processing into three steps: point cloud sampling, point cloud filtering, and point cloud registration. In this study, three different point cloud algorithms were selected for processing in different steps, and under the premise of ensuring the basic uniformity of data volume, key parameters were selected for analysis to find the data processing algorithm with the highest accuracy. Firstly, point cloud sampling and point cloud filtering were carried out on the data of the two stations, and each of them can had nine combinations. Then, the two station clouds were aligned and combined, and 81 kinds of registration method were obtained. Different registration algorithms affected the final data accuracy in various ways, and studying these impacts helped optimize the processing workflow. A detailed introduction and evaluation of point cloud data analysis are presented in the subsequent sections. The point cloud data processing framework is shown in Figure 1.

This framework offers a comprehensive evaluation of a range of point cloud processing methods. By systematically assessing the effects of different algorithm combinations, it can inspire new algorithmic innovations. Additionally, it provides a higher standard reference model for point cloud data processing, contributing to the enhancement of data processing quality in the field of point cloud technology.

### 3.2. Point Cloud Data Processing Algorithms

For the point cloud sampling stage, the Octree, Space, and Random algorithms were utilized; the filtering stage employed Gauss filtering, Radius filtering, and Statistical filtering; and for the registration stage, the FPFH (Fast Point Feature Histograms), PFH (Point Feature Histograms), and ICP (Iterative Closest Point) algorithms were used.

The Octree algorithm, by establishing an octree structure, recursively divides space into smaller cubic units from the top down, selecting representative points in each unit to reduce the total number of points [33]. In the Octree algorithm, the maximum depth of the subdivision level was set to 10. The Space algorithm, based on the principle of equal spatial division, divides the entire space into regular grids, retaining only one representative point in each grid. The selected points are used to generate a new sampled point cloud while still preserving the spatial characteristics of the original point cloud. The Random algorithm employs random sampling, randomly selecting representative points from the original point cloud data. This sampling method has the lowest time complexity and is also one of the most commonly used and effective downsampling methods, but it retains the least point cloud features. Zhu M et al. [34] compared four sampling algorithms and found that at similar sampling rates, the random downsampling method consumed the least time and was the most efficient; however, the point cloud density distribution of the sampling results was uneven and unstable.

Gaussian filtering based on the Gaussian distribution function filters noise and smoothes the point cloud by calculating the weighted average of neighboring points around each point, primarily involving the standard deviation parameter. Radius filtering sets a specific search radius around each point, involving two parameters: the minimum number of points within the neighborhood sphere and the size of the neighborhood radius. Statistical filtering removes outliers that deviate from the average distance of a point’s neighbors by more than a set standard deviation by calculating and analyzing the statistical distribution of these distances. It mainly includes two parameters: the number of K-nearest neighbors and the standard deviation multiplier. The choice of K depends on the density and noise level of the data, while the standard deviation multiplier determines which points to remove. Ren Y et al. [35] found through filtering analysis of 3D scanning point cloud data of rock surfaces that the neighborhood parameter k and standard deviation setting should neither be too high nor too low to conform to the model points of a normal distribution.

**Figure 2:**
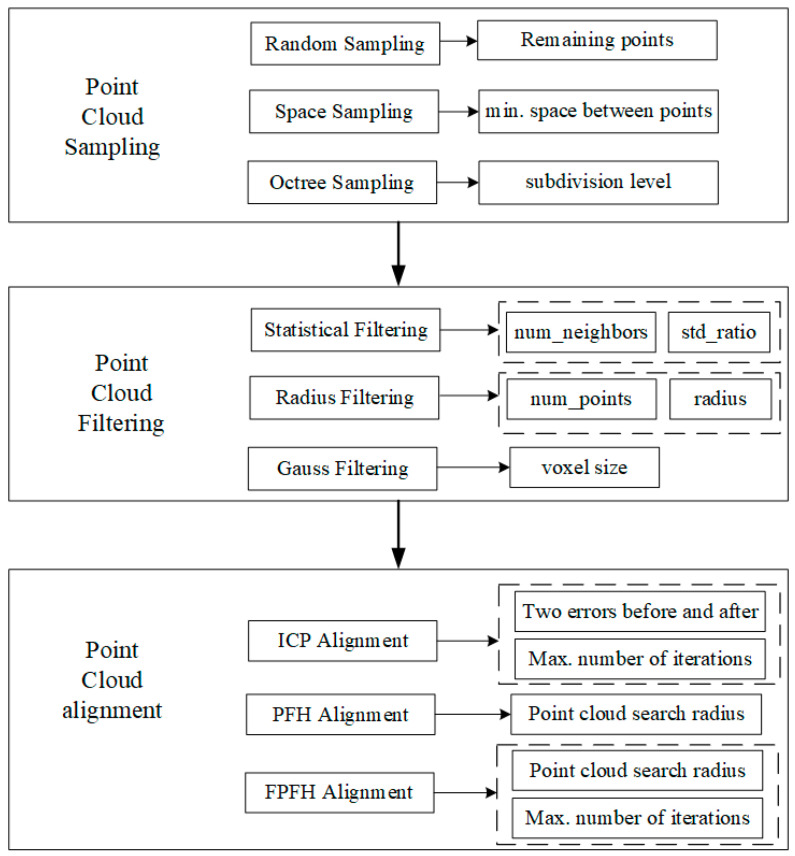
Point cloud data processing algorithm and parameters.

The ICP registration algorithm, proposed by Besl and McKay [36], is a method that finds common matching points in two overlapping point clouds and iteratively registers them based on the minimum Euclidean distance. It optimizes the rigid body transformation of the point cloud by iteratively finding matching point pairs to align the two point clouds. The PFH algorithm aims to generalize the average curvature around a point by using multidimensional histograms to encode the geometric attributes of a point’s k-nearest neighbors [37] based on the relationship between the points in the k-neighborhood and their estimated surface normality [38]. The FPFH algorithm enhances feature description by calculating the surface geometric characteristics around a point. FPFH extends the statistical analysis range by considering the neighbors of neighboring points [39], allowing for the acquisition of triplet information over a larger area when conducting feature statistical analysis in the vicinity of the source point. The key parameters of each algorithm are illustrated in Figure 2.

### 3.3. Point Cloud Data Comparison

The comparison of point cloud data was conducted to determine which algorithm combination achieved the best balance between reducing data volume, improving processing speed, and maintaining data accuracy. We adopted the following comparison strategies:

(1) Performance evaluation: conduct quantitative analysis of the results for each algorithm combination, including the rotational and translational errors along the XYZ axes before and after registration. The efficiency of the algorithms is assessed by recording their runtime and scoring.
(2) Accuracy analysis: calculate the Root Mean Square (RMS), mean distance, and standard deviation of point cloud data for accuracy analysis, evaluating the consistency and accuracy of the data after fusion.
(3) Results comparison and visualization: use graphs and visualization techniques to display the processing results of different algorithm combinations, facilitating an intuitive comparison of their performance differences.

## 4. Analysis of Point Cloud Data Processing Procedures

The algorithms in this study were implemented using the Point Cloud Library (PCL 1.8.1) in the Python (3.8.18) and Visual studio 2015 C++ programming languages. The open-source software package CloudCompare^®^ (2.13) was used to support post-processing analysis and visualization of the results.

### 4.1. Data Preparation

During the construction of the Xiong’an Library, a detailed scan of the steel truss section was conducted using the Trimble TX5 3D laser scanner (made by Trimble, Westminster, CO, USA brand). This scanner, with a range of up to 120 m and an accuracy of approximately 2 mm, is capable of capturing data at a speed of 976,000 points per second. This scanner was employed to acquire the original point cloud data required for the project. Noise compression was not applied during the scanning process, and the original point cloud data from both stations contained approximately 44 million points each. In point cloud data processing, the selection of parameters is based on the characteristics of the dataset, such as point density, noise level, and spatial distribution of the data. In this experiment, an initial comparative analysis of the parameters was first conducted to identify the optimal choice for each parameter, followed by subsequent analysis.

### 4.2. Point Cloud Parameter Settings

Point cloud sampling was performed using similar sampling rates. For Octree sampling, the subdivision level was set to 10. For Space sampling, the minimum distance between points was set to 0.1 m. For Random sampling, the number of points in the point cloud was fixed at 150,000. Based on the downsampled data, subsequent point cloud filtering and registration processes were conducted. The point cloud data volume after the sampling and filtering processes is summarized in Table 2.

Table 2 presents the point cloud data volume after sampling and filtering at each stage. The first column lists the two point clouds from stations 1 and 2, respectively. The second column indicates the different sampling algorithms selected, while the third column shows the filtering algorithms used. The fourth column displays the original point cloud data volume before processing. The fifth column shows the data volume after applying the corresponding combination of sampling and filtering algorithms. Finally, the last column provides the denoised point clouds generated from the processing.

During the filtering process, different filtering techniques require different parameter settings, and the range for each parameter is not fixed. Therefore, the initial determination of the filtering algorithm parameters was conducted to suit the data of the project. Gaussian filtering was applied with a voxel size of 0.07 m. For Radius filtering, the number of points (num_points) was set to 50, and the radius was set to 1 m. In Statistical filtering, point cloud visualization and analysis determined the number of K-nearest neighbors as 10 and the standard deviation multiplier as 0.5.

In the ICP registration algorithm, the error size before and after registration was set to 0.01 m, with a maximum of 100 iterations. In the PFH registration algorithm, the point cloud search radius was set to 0.01 m, keeping other parameters unchanged. In the FPFH registration algorithm, the point cloud search radius was set to 0.02 m, also with a maximum of 100 iterations. The detailed point cloud algorithm and parameter selection are shown in Table 3.

In registration algorithms, excessive point cloud data volume can significantly prolong computation time, preventing timely and effective feedback. Therefore, sampling of the point cloud was conducted before registration to ensure higher comparability of data during the registration process.

## 5. Analysis of Point Cloud Data Processing Results

The point cloud data was analyzed according to the different results of point cloud sampling, point cloud filtering, and point cloud registration.

### 5.1. Performance Evaluation

A quantitative analysis was conducted for the results of each algorithm combination, including rotational and translational errors along the XYZ axes before and after registration, and the runtime and scoring of the registration algorithm were recorded.

The 27 sets of data processed by the ICP registration algorithm were grouped. The 27 sets were sequentially numbered according to different processing methods, as shown in the following table. The first row shows the sampling algorithm chosen, and the second and third rows identify the filtering algorithm used at each station. The fourth row presents the registration algorithm chosen, and the fifth row is the corresponding grouping. The Radius, Statistical, and Gaussian filtering algorithms were abbreviated as R, S, and G, respectively. The same approach applies to the PFH and FPFH algorithms, with the corresponding changes made to the registration algorithms. The specific groupings are shown in Table 4.

In the ICP registration algorithm, analysis of rotational and translational errors along the XYZ axes revealed consistent patterns. Specifically, the mean squared rotation error and translation error were used as evaluation metrics. Group 16 exhibited the most favorable error fitting according to these metrics, characterized by smaller rotation and translation errors compared to other groups.

Similarly, in the PFH algorithm, rotational and translational errors displayed discernible patterns. Group 2 demonstrated the best error fitting, as determined by the lowest mean squared rotation and translation errors among all groups.

**Figure 3:**
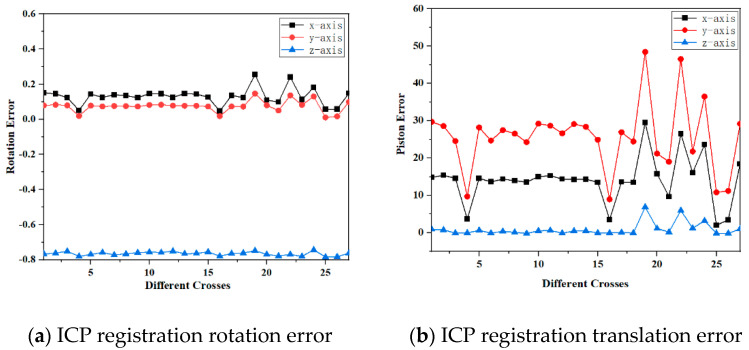

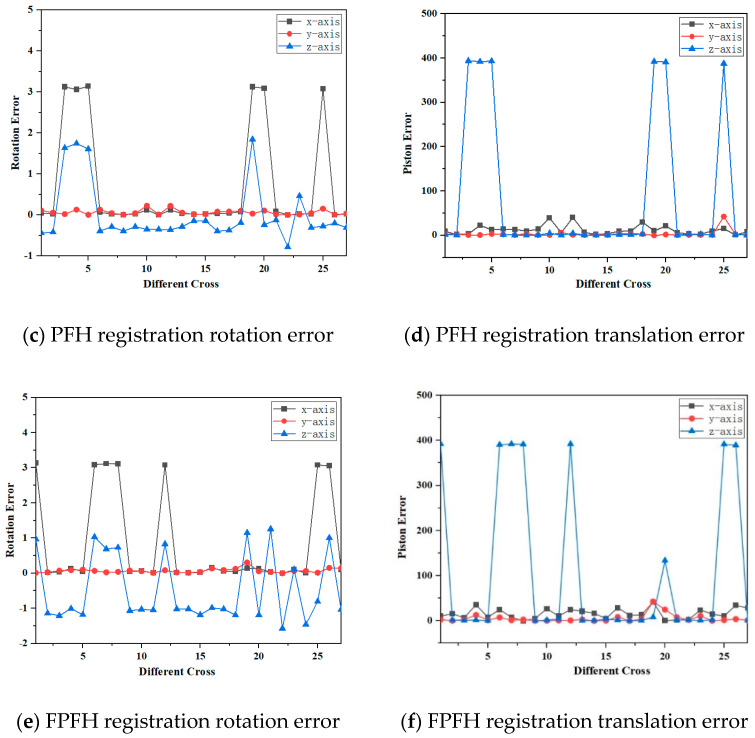
Point cloud registration rotation error and translation error.

Conversely, the FPFH algorithm exhibited less pronounced patterns in rotational and translational errors. Nevertheless, after a comprehensive evaluation, Group 22 emerged with the most favorable error fitting. The error results for all subgroups are shown in Figure 3. Further analysis focused on elucidating the specific characteristics of this group’s data.

**Figure 4:**
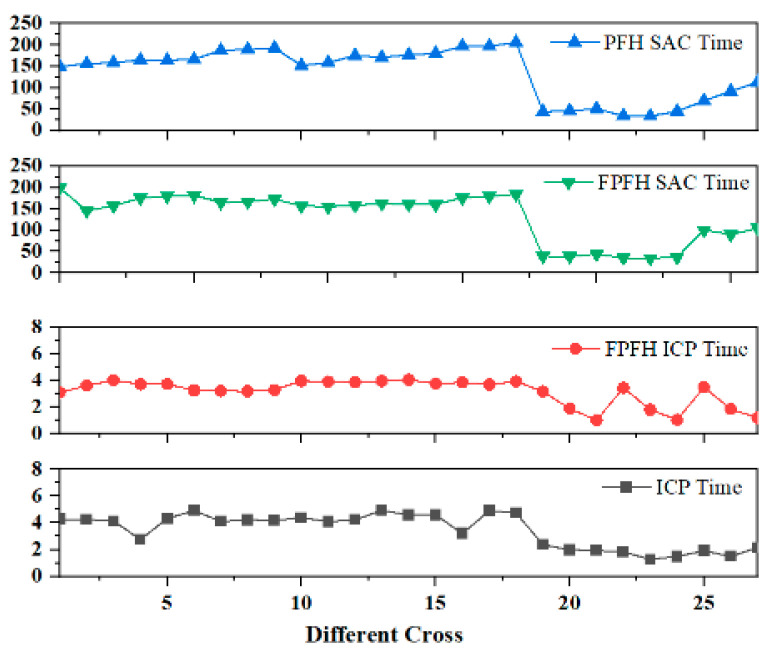
Registration time.

The results in terms of algorithmic registration time are shown in Figure 4. It was generally observed that Groups 19 to 27 had relatively shorter registration times. This is because these groups employed the Random algorithm for point cloud sampling, a method that randomly selects points from the point cloud as the result of downsampling. Although this approach is simple and fast, it does not take into account factors such as the distribution and geometric characteristics of the points.

**Figure 5:**
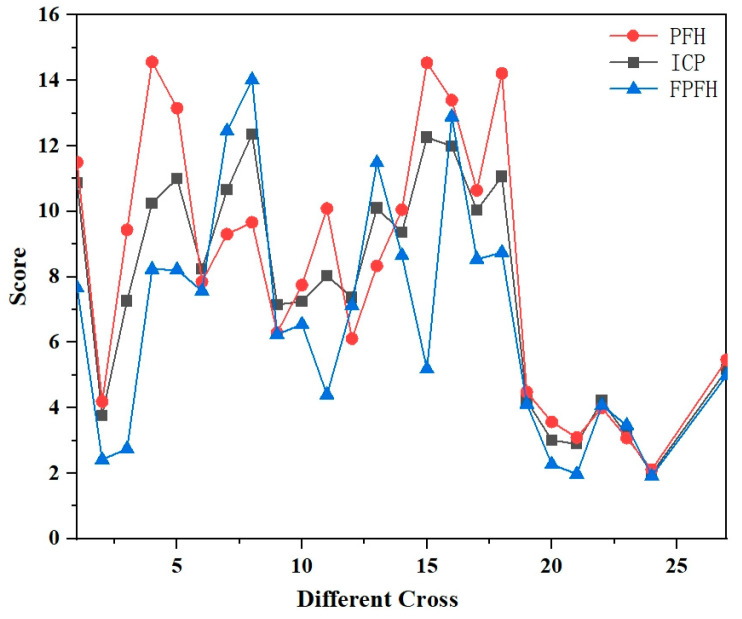
Registration score.

Registration scoring: In point cloud registration using the Sample Consensus Initial Alignment (SAC-IA) algorithm from the PCL, the fitness score is a key metric for assessing the quality of registration. It is obtained by calculating the sum of squared distances between each point in the source point cloud and its nearest neighbor in the target point cloud. Consequently, a lower score indicates a higher quality of registration, as it signifies that the points in the source point cloud are closer to their corresponding points in the target point cloud. The results of the registration score are shown in Figure 5. Upon analyzing the score results, it was observed that the results of registration after Random sampling followed by Statistical and Gaussian filtering tended to be comparatively better. The use of Gaussian filtering may lead to a sudden change in the score.

Taken together, there was no fixed “good” combination of scores in the score analysis, as it is highly dependent on the specific application scenario, the size and density of the point cloud, and other parameter settings of the registration algorithm. To more accurately assess the quality of registration, it is often necessary to consider the requirements of the actual application or calculate other statistical metrics, such as the Root Mean Square (RMS).

An in-depth analysis was conducted on Group 16 registered using the ICP algorithm, Group 2 registered using the PFH algorithm, and Group 22 registered using the FPFH algorithm.

### 5.2. Precision Analysis

The accuracy of registration was evaluated using known benchmark data. Statistical methods were employed to analyze the precision of the results, calculating the Root Mean Square (RMS), mean distance, and standard deviation of the point cloud data. The RMS Error is currently the most commonly used metric to describe the accuracy of registration, effectively representing the specific errors between models after registration. The Root Mean Square (RMS) is typically used to measure the average magnitude of a set of values. The calculation formula is as follows:

$$RMS=\sqrt{\frac{1}{N}\Sigma_{i=1}^{N}y_{i}^{2}}$$

In this formula, N represents the total number of observation points, $y_{i}$ is the actual value of the *i*-th observation point, and ${\hat{y}}_{i}$ is the predicted value of the *i*-th observation point.

**Figure 6:**
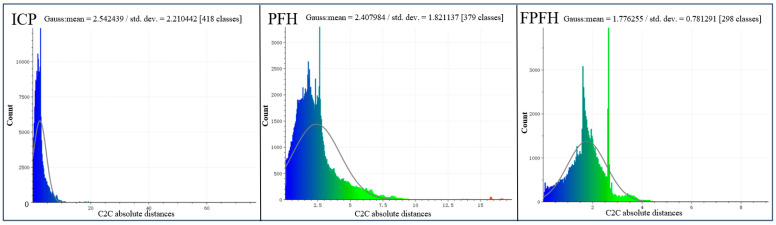
Point cloud registration C2C absolute distance.

Mean distance is the average value of the distance between all pairs of points in the cloud. The standard deviation of the distance indicates the dispersion of the distance values around the mean value. Through these two values, it is possible to comprehend the average level of distance between points in the entire dataset and the range of variations in these distances. Point cloud registration C2C absolute distance results are shown in Figure 6.

For Group 16 registered using the ICP algorithm, the cloud-to-cloud (C2C) distance of the point cloud was calculated. The histogram indicates that most data points were concentrated near smaller distance values, demonstrating that most points in both sets of point clouds were very close to each other, which indicates good alignment. For the C2C absolute distance, Gauss mean = 2.542 and Gauss standard deviation = 2.21. The final RMS after the registration of the two sets of data was 1.299.

For Group 2 registered using the PFH algorithm, the data followed a bell-curve distribution that is typical of a normal distribution, but with a slight skew to the right. In the C2C absolute distance, Gauss mean = 2.408 and Gauss standard deviation = 1.821. The final RMS after the registration of the two sets of data was 0.802.

For Group 22 registered using the FPFH algorithm, the data distribution showed a strong peak at lower distance values, indicating that many points between the two clouds were very close to each other. The Gaussian curve fitting did not match the distribution well, especially in the tail area. This suggests that the actual distance distribution might not be normal and could have skewness or outliers. In the C2C absolute distance, Gauss mean = 1.776 and Gauss standard deviation = 0.781. The final RMS after the registration of the two sets of data was 0.794.

### 5.3. Comparison and Visualization of Results

**Figure 7:**
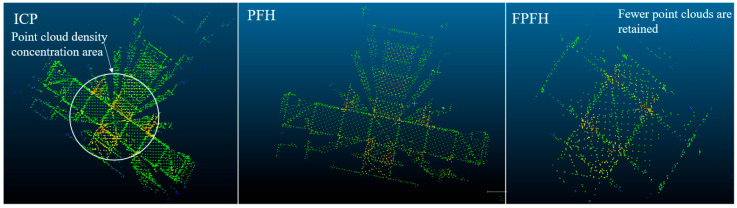
Registration visualization.

The visualization results of the registration are shown in Figure 7. The color gradient from blue to red indicates the point cloud density from sparse to dense, with red areas denoting regions of higher point cloud density and blue areas signifying regions of lower point cloud density. The visualization result of Group 16 registered using the ICP algorithm was the best, although there were still some distant noise points. The data retained for Group 22 registered using the FPFH algorithm were the worst, resulting in a poorer visualization effect.

**Figure 8:**
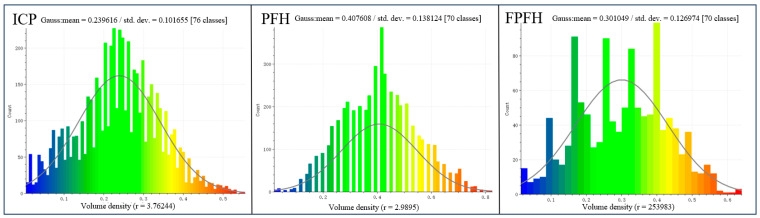
Volume density.

The point cloud volume density distribution is shown in Figure 8, with the x-axis indicating the range of volume density and the y-axis indicating the number of occurrences of the point cloud data. The mean value of the aligned data in Group 16 of the ICP algorithm registration was approximately 0.240, with a standard deviation of 0.102. The Gaussian fitting curve was generally consistent with the data distribution in the histogram, but with a slight deviation in the tail region, indicating that there were some skews or outliers in the actual data distribution. Group 2 of the Gaussian distribution curve fitted by the PFH algorithm shows the expected distribution when the distribution is normal, and the mean value of the data was 0.408 with a standard deviation of 0.138. Group 22 of the data fitted by the FPFH algorithm had a mean value of about 0.301 with a standard deviation of 0.127.

Based on a comprehensive analysis of the above, the concentration of data, and the quality of the fit as the criteria, the point cloud data from Group 16 registered using the ICP algorithm showed the best results. Its Gaussian fitting curve aligned well with the histogram data, having a lower mean and smaller standard deviation. This indicates that the data points were more tightly clustered around the central value, and the overall distribution consistency was higher. Utilizing the results processed from this group for construction quality control will more comprehensively reflect the actual situation at the construction site.

## 6. Conclusions

This study proposes a comparison framework based on point cloud data processing algorithms, which can effectively evaluate the performance of different algorithm combinations and explore optimal point cloud processing based on registration accuracy and data fidelity. By optimally combining sampling, filtering, and alignment algorithms, the efficiency and accuracy of large-scale point cloud data processing for large buildings can be improved. This study determined the parameter choices for point cloud data processing and conducted a detailed comparative analysis through three major aspects: performance evaluation, accuracy analysis, and results comparison and visualization. It determined the optimal combination of results for different registration algorithms under the current experimental conditions. The comparative framework of this study is important for understanding the mechanisms and applicability scenarios of different algorithms in point cloud data processing.

The research presented in this paper demonstrates that a rational combination of downsampling, filtering, and registration algorithms can significantly improve the efficiency of point cloud data processing while meeting the accuracy and completeness requirements of engineering data. The optimization scheme proposed in this study can significantly improve the processing efficiency of large-scale point cloud data, especially in terms of finding an efficient combination of algorithms that meet the requirements of accuracy and data fidelity. This has direct application value for engineering fields that need to process large amounts of point cloud data, such as architectural engineering, urban planning, and UAV surveying.

Notably, the findings of this study are not only reflected in the improvement of data processing efficiency but also reveal the applicability of different algorithm combinations in different scenarios. For example, the combination of Octree sampling and ICP alignment can significantly reduce the processing time while ensuring data integrity, while the combination of Gauss filtering and FPFH alignment excels in detail preservation. This in-depth exploration of algorithm combinations helps us to better understand how to choose the most appropriate processing flow according to specific needs, so as to achieve a balance between efficiency and accuracy.

However, there were some limitations to this study. Firstly, due to the limited performance of the computing equipment used, there was excessive data compression in the sampling part of the point cloud data processing, which impacted the detail and accuracy of the results. Secondly, the approach of using a single fixed value for parameter selection may have to some extent limited the performance of the algorithms. Although this study identified the superior performance of certain algorithm combinations under the current experimental conditions, these results do not imply that the combination is optimal in all scenarios. The effectiveness of point cloud processing is likely to vary significantly depending on the characteristics of the dataset, changes in application scenarios, and different task requirements. The current experimental results are based on specific point cloud datasets and application contexts, and thus the “optimal algorithm combination” should be understood as the best solution under these particular conditions. Further validation is required if this combination is to be applied in different scenarios.

Future research directions should further explore the automation of point cloud data processing in conjunction with the range of parameter choices based on this study and improve the performance and adaptability of point cloud processing algorithms using deep learning. 

## Figures and Tables

**Table 2:** Amount of point cloud data after point cloud sampling and filtering processes.

	Sampling Algorithm	Filtering Algorithm	Primordial Point Cloud	Post-processing Point Cloud	Noise Reduction Point Cloud
1	Octree	Gauss	150,554	145,475	5079
		Radius		147,864	2690
		Statistical		147,825	2729
	Space	Gauss	184,867	174,548	10,319
		Radius		178,296	6571
		Statistical		180,137	4730
	Random	Gauss	150,000	10,394	94,349
		Radius		139,606	10,394
		Statistical		88,391	61,609
2	Sampling Algorithm	Filtering Algorithm	Primordial Point Cloud	Post-processing Point Cloud	Noise Reduction Point Cloud
	Octree	Gauss	157,497	151,288	6209
		Radius		150,140	7357
		Statistical		151,479	6018
	Space	Gauss	178,520	169,433	9087
		Radius		171,207	7313
		Statistical		168,513	10,007
	Random	Gauss	150,000	52,614	97,386
		Radius		140,263	9737
		Statistical		80,048	69,952

**Table 3:** Point cloud algorithm parameter selection.

Sampling Algorithm	Parameter Details	Filtering Algorithm	Parameter Details	Registration Algorithm	Parameter Details
Octree	subdivision level: 10	Gauss	voxel size: 0.07	ICP	Two errors before and after: 0.01 m
					Maximum number of iterations: 100
Space	min. space: 0.1 m	Radius	num_points: 50	PFH	Point cloud search radius: 0.01 m
			Radius: 1 m		
Random	Remaining points: 150,000	Statistical	num_neighbors: 10	FPFH	Point cloud search radius: 0.02 m
			std_ratio: 0.5		Maximum number of iterations: 100

**Table 4:** Grouping of point cloud algorithms.

Sampling		Octree									Space									Random								
Filtering	1	R			S			G			R			S			G			R			S			G		
	2	R	S	G	R	S	G	R	S	G	R	S	G	R	S	G	R	S	G	R	S	G	R	S	G	R	S	G
Registration		ICP																										
Grouping		1	2	3	4	5	6	7	8	9	10	11	12	13	14	15	16	17	18	19	20	21	22	23	24	25	26	27

Note: The Radius, Statistical, and Gaussian filtering algorithms were abbreviated as R, S, and G, respectively.

## Data Availability

Data are contained within the article.
